# Hepatocyte Intrinsic Innate Antiviral Immunity against Hepatitis Delta Virus Infection: The Voices of Bona Fide Human Hepatocytes

**DOI:** 10.3390/v16050740

**Published:** 2024-05-08

**Authors:** Yein Woo, Muyuan Ma, Masashi Okawa, Takeshi Saito

**Affiliations:** 1 Division of Gastrointestinal and Liver Diseases, Department of Medicine, Keck School of Medicine, University of Southern California, Los Angeles, CA 90033, USA; 2 R&D Department, PhoenixBio USA Corporation, New York, NY 10006, USA; 3 USC Research Center for Liver Diseases, Los Angeles, CA 90033, USA; 4 Department of Molecular Microbiology and Immunology, Keck School of Medicine, University of Southern California, Los Angeles, CA 90033, USA; 5 Department of Pathology, Keck School of Medicine, University of Southern California, Los Angeles, CA 90033, USA

**Keywords:** hepatitis delta virus (HDV), hepatitis B virus (HBV), human hepatocyte, humanized liver chimeric mouse, humanized experimental model

## Abstract

The pathogenesis of viral infection is attributed to two folds: intrinsic cell death pathway activation due to the viral cytopathic effect, and immune-mediated extrinsic cellular injuries. The immune system, encompassing both innate and adaptive immunity, therefore acts as a double-edged sword in viral infection. Insufficient potency permits pathogens to establish lifelong persistent infection and its consequences, while excessive activation leads to organ damage beyond its mission to control viral pathogens. The innate immune response serves as the front line of defense against viral infection, which is triggered through the recognition of viral products, referred to as pathogen-associated molecular patterns (PAMPs), by host cell pattern recognition receptors (PRRs). The PRRs–PAMPs interaction results in the induction of interferon-stimulated genes (ISGs) in infected cells, as well as the secretion of interferons (IFNs), to establish a tissue-wide antiviral state in an autocrine and paracrine manner. Cumulative evidence suggests significant variability in the expression patterns of PRRs, the induction potency of ISGs and IFNs, and the IFN response across different cell types and species. Hence, in our understanding of viral hepatitis pathogenesis, insights gained through hepatoma cell lines or murine-based experimental systems are uncertain in precisely recapitulating the innate antiviral response of genuine human hepatocytes. Accordingly, this review article aims to extract and summarize evidence made possible with bona fide human hepatocytes-based study tools, along with their clinical relevance and implications, as well as to identify the remaining gaps in knowledge for future investigations.

## 1. Introduction

Hepatitis D Virus (HDV) is a satellite pathogen of Hepatitis B Virus (HBV) as it requires HBV surface antigen (HBsAg) for virion assembly [1,2]. Therefore, HDV infection only occurs in individuals with concurrent HBV infection [3]. There are two different modes of HBV–HDV infection: (1) co-infection, in which HBV and HDV simultaneously establish infection, and (2) superinfection, wherein HDV infects individuals who are chronically infected with HBV. Currently, 350 million people worldwide are chronically infected with HBV, of whom more than 10% are estimated to be coinfected with HDV [4,5,6]. However, this number might be underestimated due to gaps in the epidemiological data, particularly in resource-limited countries [7].

HDV infection has been regarded as the most severe type of hepatitis virus due to the increased risk of developing fulminant hepatitis during acute infection, as well as the rapid disease progression to cirrhosis and liver cancer in chronic infection [8]. Both co- and superinfection of HBV–HDV are associated with substantial morbidity and mortality [8,9]. However, the mechanisms through which HDV infection leads to accentuated liver inflammation, rapid progression of fibrosis, and the oncogenic transformation of hepatocytes remain largely elusive.

One of the key determinants of viral pathogenesis is the intricate interplay between the virus and the immune system, which includes intracellular and cellular innate immunity as well as adaptive immunity. The intracellular innate immunity serves as the first line of defense against viral infections, orchestrating a coordinated response through the recognition of viral infections and the activation of downstream signaling cascades that govern the induction of antiviral genes, referred to as interferon-stimulated genes (ISGs) and interferons (IFNs) [10,11,12]. ISGs comprise more than 400 genes, the expression and inducibility of which vary considerably between cell types [13,14,15,16]. The type and magnitude of IFN production in infected cells also differ significantly among cell types [17]. Therefore, the role of the IFN system, encompassing ISG induction and IFN production, varies greatly depending on the disease context, the immunogenicity of the pathogens, and the affected organs and cell types [14,18].

Accordingly, it is imperative to employ physiologically relevant research tools in order to advance our comprehension of the significance of the IFN system in the regulation of HDV infection. The elucidation of virus–host interactions and the consequential activation of the IFN system with proper research tools is crucial not only for deciphering the pathogenic mechanisms underlying HDV-induced liver disease but also for developing effective therapeutic strategies.

In the human liver, hepatocytes, the target of HDV infection, are terminally differentiated and hence non-proliferating, fulfilling their specialized functions, commonly referred to as “liver function”, such as detoxification, bile secretion, deamination, and the metabolism–synthesis–storage of nutrients [19]. Terminally differentiated human hepatocytes (hereafter referred to as human hepatocytes), such as primary human hepatocytes (PHHs) and humanized liver chimeric mice (HLCM)-derived human hepatocytes (HLCM-HHs), are the most representative experimental platforms for in vitro studies [20]. With recent advancements in culture methods, in vitro cultured human hepatocytes maintain their genuine characteristics over the long term, such as the gene expression profile, morphological appearance, and functionality [21]. All of these attributes combined distinguish bona fide human hepatocytes from hepatoma cell lines such as HepG2, Huh7, and HepaRG cells [22,23,24].

With regards to the in vivo model, the HLCM system, also known as chimeric mice with humanized livers, is thus far deemed the most physiologically relevant experimental platforms for in vivo studies of human liver biology and diseases [21,25,26,27,28]. While conventional mouse models have been extensively utilized in a wide range of biomedical studies, the significant limitations and obstacles resulting from interspecies differences between humans and rodents have been increasingly recognized. These include, but are not limited to, the lack of susceptibility to HBV and HDV infection in rodents, while these pathogens establish efficient life cycles and persistent infection in HLCM [29,30].

These modern study tools, human hepatocytes and the HLCM system, have been increasingly employed for studies of viral hepatitis pathogenesis, virus–host interaction, and innate antiviral response, as well as therapeutic intervention [25,27,28]. This review article summarizes the cumulative knowledge on the significance of the innate antiviral response against HDV infection, with a particular focus on insights gleaned through the use of human hepatocytes (PHHs, HLCM-HHs), and HLCM systems.

## 2. HDV Infection and Host Response in Genuine Human Hepatocytes

Humans and chimpanzees are the only natural hosts of HDV [31]. The narrow host range is largely due to the interspecies differences in the amino acid sequence of sodium taurocholate cotransporting polypeptide (NTCP), a cell surface protein that facilitates viral entry via interaction with HBsAg on the viral envelope [32,33,34,35]. Hence, human hepatoma cells, such as HepaRG cells and NTCP-overexpressing HepG2 cells, have been the mainstay research platform, and observations made with these tools constitute the basis of our current understanding of HDV infection [36]. These models, however, exhibit fundamental limitations since they possess only marginal similarity to the original primary cells; both the resting phenotype and antiviral response, including its potency and pattern, are substantially altered in comparison to bona fide human hepatocytes such as PHHs and HLCM-HHs [14,15,18,21,22,24].

To date, there exists a scarcity of information regarding the innate antiviral response of human hepatocytes, specifically in the context of HDV infection. Two recent studies have demonstrated that HDV mono-infection, which allows only a single entry and subsequent intracellular replication, is capable of establishing efficient replication in human hepatocytes and persisting for at least two months [29,36]. Of important note, the efficiency of viral replication in non-proliferating, terminally differentiated human hepatocytes is considerably greater than that in hepatoma cell lines [29,36]. This disparity is likely attributed to the well-preserved characteristics of genuine hepatocytes, including, but not limited to, the abundant expression of NTCP [21,29], emphasizing the criticalness of the choice of experimental platforms to be employed.

HDV infection of human hepatocytes triggers a robust and sustained induction of ISGs throughout the course of infection, with a magnitude comparable to that induced by treatment with therapeutic doses of type I IFN [29]. It is possible that this phenomenon is not exclusive to HDV genotype 1a, which is the strain frequently utilized in molecular virological studies, given that comparable degrees of ISG induction are observed irrespective of the genotype or strain [37].

In contrast, HBV mono-infection, which has been known as a stealth virus in the activation of the hepatic IFN system [38], exhibits a negligible impact on the expression abundance of ISGs [29]. Accordingly, HBV–HDV infection of human hepatocytes, both co- and superinfection, results in the activation of the IFN system, in which the extent of the ISG induction is notably greater in superinfection compared to co-infection. This difference likely arises from the immediate availability of a greater number of HBV-infected hepatocytes, which would facilitate the rapid and robust establishment of the HDV life cycle, ultimately resulting in efficient tissue-wide spread. These observations, using in vitro cultured human hepatocytes systems, indicate that HDV is highly immunogenic in the activation of hepatocyte intrinsic antiviral response [29,36]. In vivo studies with HLCM provided an additional layer of evidence, wherein chronic HBV–HDV infection is coupled with a notable increase in the expression of ISGs in the liver tissue, and more specifically, in human hepatocytes [29,39].

However, despite the potent activation of the innate antiviral response, the HDV life cycle continues to persist in infected hepatocytes. This phenomenon could be explained through at least three potential scenarios: (1) the hepatocytes intrinsic innate antiviral response is not yet at a magnitude sufficient to control HDV infection, (2) HDV possesses a mechanism to evade or abrogate the extent of the innate antiviral response activation in hepatocytes; and/or (3) HDV is equipped with a high degree of resistance to the antiviral properties of ISGs.

## 3. Viral Sensors of Hepatocytes in the Recognition of HDV Infection

The antiviral immune response begins when infected cells sense the presence of pathogen-associated molecular patterns (PAMPs) via pattern recognition receptors (PRRs) [40,41]. Hence, the first step in understanding the antiviral response against HDV infection is to decipher how hepatocytes, the sole parenchymal cell type of the liver and a non-immune cell, sense the PAMPs of HDV [40]. The interactions between PRRs and PAMPs are determined by the expression pattern of PRRs in each cell type and the unique biochemical features of PAMPs. The expression profile of PRRs in hepatocytes has been controversial, especially for TLRs, which are responsible for sensing PAMPs in the extracellular space and/or in the endosome [42,43]. This confusion arises in part from the indiscriminate use of the term “hepatocytes” to describe cell types utilized in published studies, which often include not only bona fide human hepatocytes but also liver cancer cell lines and stem cell-derived hepatocytes with uncertain levels of cell maturation. In human hepatocytes, despite being detectable at the level of the transcript, the expression abundance of TLRs has been considered insignificant, as evidenced by the absence of biologically significant responses to their specific ligands, particularly in the activation of the IFN system [42,43]. Accordingly, to date, there have been no studies suggesting the involvement of TLRs in the activation of antiviral immune responses against HDV in hepatocytes.

In contrast, an alternative class of PRRs responsible for sensing viral PAMPs within the cytosol has been believed to play a major role in the activation of intracellular antiviral immunity in HDV-infected hepatocytes. Retinoic acid-inducible gene I (RIG-I)-like receptors (RLRs) consisting of three members, RIG-I, melanoma differentiation-associated gene 5 (MDA5), and laboratory of genetics and physiology 2 (LGP2), belong to the DEAD-box RNA helicase protein superfamily [40,44]. RLRs constantly scan RNA species and RNA:DNA hybrids in the cytosol to identify their potential ligands, and upon recognition of which leads to the activation of a signaling cascade to the induction of ISGs as well as IFNs [45]. RIG-I and MDA5 play largely non-redundant roles by sensing distinct biochemical features of nucleic acids, while LGP2 serves as either a positive or negative regulator of RIG-I and MDA5 [41,46,47].

It was discovered that the activation of the hepatocytic IFN system during HDV infection is markedly reduced with shRNA-based gene silencing of MDA5, while RIG-I silencing showed no such effect [36]. This observation has formed the basis for the notion that MDA5 serves as the primary PRR responsible for sensing HDV-PAMPs and subsequently inducing ISGs as well as type I and III IFNs. However, it should be noted that this presumption was established based on a single time point experiment conducted with HepaRG cells, a hepatoma cell line. Therefore, it is plausible that the significance of MDA5 could differ in human hepatocytes due to variations in the relative abundance to RIG-I, in addition to the difference in viral replication efficiency among different cell types. In addition, emerging evidence reveals that the mechanism and potency of RLR activation are profoundly influenced by the cellular metabolic status [48,49], emphasizing the necessity of further investigations with physiologically relevant experimental formats.

**Figure 1:**
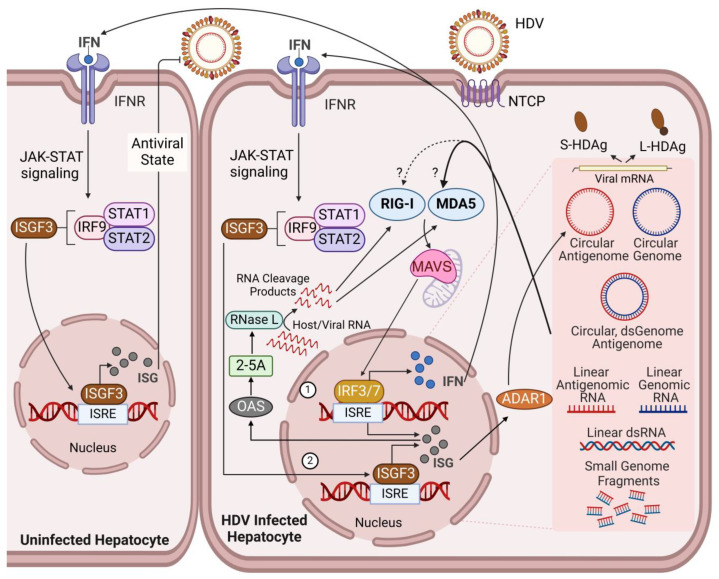
Overview of the hepatic IFN system in the regulation of HDV infection in human hepatocytes: current understanding and gaps in knowledge. HDV enters hepatocytes through the interaction between its HBsAg and the host cell surface protein NTCP. Replication occurs in the nucleus, generating various viral RNA species. Currently, MDA5, one of the RLHs, and to a lesser extent, RIG-I, are considered key PRRs that sense HDV PAMPs (vRNA species). The interaction between RLHs and vRNA species triggers the activation of the MAVS-IRF3/7 pathway and induces ISGs and IFNs (1). ADAR1, an ISG, facilitates the HDV life cycle by introducing a point mutation enabling L-HDAg production, functioning as a proviral host factor. OAS, another ISG, activates the RNaseL pathway via the production of ppp2′-5′A, which in turn produces RIG-I and MDA5 ligands through cleaving vRNA and host RNA species. Therefore, both RIG-I and MDA5 are expected to play a role in the induction of ISGs and IFNs in HDV infection. IFNs, predominantly type III IFNs, secreted from the infected hepatocytes act on both infected and infection-naïve hepatocytes to induce ISGs via activation of Jak-STAT signaling cascades (2); thereby serving as the second wave of the antiviral response in the infected cells as well as establishing a tissue-wide antiviral state in the liver. Despite these sophisticated innate antiviral responses, the hepatocyte intrinsic IFN system is incapable of halting HDV infection due to HDV’s high resistance to the antiviral properties of ISGs and the establishment of cellular IFN refractoriness resulting from constitutive exposure to IFNs.

The HDV life cycle involves the rolling circle replication mechanism [50]; thus, infected cells harbor multiple types of viral RNA (vRNA) species, such as linear genomic/antigenomic intermediates, dsRNA replication intermediates due to the intramolecular base pairing of the HDV genome and antigenome, viral mRNA, small genome fragments resulting from the ribozyme cleavage, as well as the circular genome and antigenome (Figure 1). While it remains entirely unclear which vRNA species have the most significant impact on the activation of the innate antiviral response in hepatocytes, it is conceivable that the replication intermediate dsRNA plays a predominant role, as MDA5 preferentially senses long dsRNA [51]. Of important note, the total and relative abundance of each HDV vRNA species are expected to differ depending on the stage of infection, such as the super acute, subacute, and persistent infection phases. Hence, the significance of MDA5 as the PRR of HDV infection might not remain consistent throughout the course of infection.

In addition, the potent and persistent upregulation of 2-5-oligoadenylate synthase (OAS), an interferon-stimulated gene (ISG), throughout the course of HDV infection in human hepatocytes strongly suggests the subsequent activation of the RNaseL pathway (Figure 1) [29], which leads to the production of potential ligands for RLR activation. OAS facilitates the formation of 2,5-linked phosphodiester bonds to synthesize adenosine polymer (pppA(2′p5′A)n), which consequently acts as a second messenger to activate RNaseL. The activated RNaseL cleaves both viral and host RNA in the cytosol as well as in the nucleus. These cleavage products have been shown to serve as ligands for RIG-I and MDA5 [52,53].

Based on the above, it remains elusive whether the activation of the IFN system in HDV-infected hepatocytes is solely reliant on the MDA5 signaling pathway. Further comprehensive studies using physiologically relevant experimental platforms, genuine human hepatocytes, PHHs and HLCM-HHs, together with biochemical and genetic approaches, are necessary.

## 4. Role of the Hepatocytic IFN System in the Regulation of HDV Infection

HDV infection of human hepatocytes, whether in vitro or in vivo, triggers a robust and sustained activation of the IFN system [29,39], which is comparable in magnitude to that induced by type I IFN treatment [29]. This activation results in the induction of over 100 ISGs in hepatocytes, which are expected to cooperatively suppress the efficiency of the viral life cycle [29,54,55,56,57]. However, emerging evidence suggests that HDV is highly resistant, or irresponsive, to the antiviral action of ISGs [29], which is in accordance with the inadequate clinical efficiency of IFN therapy [9,58,59].

In general, upon viral infection, the interaction between viral PAMPs and PRRs initiates the first wave of the antiviral response, the induction of ISGs, through the activation of the MAVS-IRF3/7 pathway (Figure 1) [40,41]. This event results in not only the induction of ISGs but also IFNs, predominantly type III IFNs, in the case of human hepatocytes [29,60,61]. Type III IFNs (IFN-λ1, -λ2, and -λ3), also known as IL-28/29, act on epithelial cells such as hepatocytes, triggering the activation of the Jak-STAT signaling cascade [62,63]. This process facilitates the formation of the transcription factor, ISGF3, which consists of STAT1, STAT2, and IRF9. ISGF3 governs the induction of a largely redundant set of genes—ISGs—that are regulated by the MAVS-IRF3/7 pathway (Figure 1). Therefore, through an autocrine mechanism, the type III IFNs secreted from infected hepatocytes act as the second wave of the ISG induction machinery. However, in the setting of persistent activation of the IFN system, this highly sophisticated antiviral program could exhibit detrimental effects. This is because prolonged exposure to IFNs, either in a paracrine or autocrine manner, establishes a state of refractoriness [29,64,65]. In general, this mechanism is viewed as a safety-valve system to prevent the forward-feed amplification of the IFN system, as excessive activation would lead to the activation of programmed cell death and/or autoimmune diseases [66,67,68]. In HDV infection, the robust and sustained production of type III IFNs, and the consequent autocrine action, renders infected hepatocytes insensitive, or perhaps unresponsive, to IFNs [29]. Hence, the persistent and potent activation of the hepatic IFN system by HDV paradoxically protects HDV, at least to a certain extent, from the intracellular antiviral immunity through the establishment of IFN refractoriness. Regarding the mechanisms through which HDV establishes IFN refractoriness in human hepatocytes, the upregulation of USP18 and ISG15 [29]—both ISGs and well-accepted negative regulators of IFN signaling [69,70]—plays central roles rather than other negative regulators, such as the suppressor of cytokine signaling (SOCS) protein family.

The ineffectiveness of IFNs in suppressing HDV infection could also be attributed to viral factors. In our in vitro assessment conducted with human hepatocytes stably infected with HBV–HDV, treatment with PegIFNα-2a (ranging from 0 to 250 ng/mL) (note: the peak serum concentration in humans after the first dose of 180 µg/60 kg body weight is approximately 12.5 ng/mL) [71] is found to effectively achieve HBV suppression in a dose-dependent manner [29]. In contrast, it fails to suppress HDV replication regardless of the dosage, indicating that the sensitivity to the antiviral properties of type I IFN differs between each pathogen, with HDV exhibiting much lower sensitivity compared to HBV. Moreover, a similar phenomenon is observed in our unpublished study, where HDV showed a lack of sensitivity to both type II IFN (IFN-γ) and type III IFNs (IFN-λ). Moreover, in vivo studies with HLCM with HBV–HDV superinfection revealed consistent findings with those observed in human hepatocytes. The therapeutic PegIFNα-2a had a negligible impact on the replication efficiency of HDV, while demonstrating substantial antiviral efficacy in the suppression of HBV [29]. Furthermore, similar to HBV but unlike HDV, a significant antiviral response to PegIFNα-2a has also been observed in HLCM chronically infected with HCV [72]. This evidence from both in vitro and in vivo studies collectively indicates that HDV possesses a high degree of resistance to the antiviral properties of ISGs when compared with that of HBV and HCV [72,73].

It is important to note that the establishment of the HDV life cycle requires one of the ISGs, adenosine deaminase acting on RNA 1 (ADAR1), which introduces a point mutation that eliminates the stop codon of S-HDAg, resulting in the addition of 19 amino acids to generate L-HDAg [74]. This event regulates the relative abundance of S- and L-HDAg, which are necessary for genome replication and virion assembly, respectively [75,76]. Hence, the HDV activation of the PRR signaling pathway and subsequent induction of ADAR1 might represent a viral immune evasion strategy. Taken together, the currently available evidence suggests that the HDV life cycle is maintained through a delicate balance between the exploitation of ISG induction machinery, the development of IFN refractoriness in infected hepatocytes, and its high resistance to the antiviral actions of ISGs.

## 5. Impact of Hepatocytic IFN System Activation by HDV on HBV Infection

The efficiency of the viral life cycle is determined by the equilibrium between antiviral immunity and viral virulence, and persistent infection is only established when this balance tilts in favor of the invading pathogen. Accordingly, viral pathogens, especially those known to develop persistent infection, are equipped with a multitude of mechanisms that either avoid, evade, or disable antiviral immune responses. This statement also holds true for HBV infection, where several studies have demonstrated that HBV infection of human hepatocytes, whether PHHs, HLCM-HHs, or HLCM, results in minimal, and perhaps subtle, changes in ISGs expression [29,39]. Due to this phenomenon, HBV has established its characteristic as a stealth virus [38], allowing it to evade detection by the innate immune system and therefore efficiently transition to the lifelong persist infection. HBV’s capacity to evade the activation of the hepatocytic IFN system is likely a reflection of its susceptibility to the antiviral properties of ISGs [29,73]. In fact, IFN treatment, as well as the RIG-I agonist, exhibits potent antiviral activities in suppressing HBV replication in human hepatocytes [18,29,44,73,77].

Consequently, in the setting of HDV co-infection or superinfection, HBV’s replication efficiency is hindered by its sensitivity to the antiviral properties of ISGs. In both in vitro and in vivo studies, the concurrent infection of HDV results in the suppression of HBV though the activation of the hepatocytic IFN system [29,39]. This observation challenges the principle of viral interference. In general, viral interference serves as a mechanism through which the primary pathogen makes the most of its “first come, first served” position to monopolize the host cell machinery, establishing its successful viral life cycle while minimizing the threat posed by the secondary pathogen [78,79,80]. Thus, in the case of HBV–HDV infection, the conventional concept of viral interference does not apply and exhibits a paradoxical mode of viral interference wherein HDV, the superinfectant, outcompetes HBV, the primary pathogen, due to their distinctive immunogenicity and varying sensitivity to the antiviral actions of ISGs.

The burden of concurrent HDV infection is not confined to the level of innate immunity since a number of the molecules involved in the antigen presentation are also ISGs. Hence, HDV infection augments the activation of anti-HBV adaptive immunity [81]. These observations together indicate that the relationship between HBV and HDV is not mutually beneficial: only HDV takes credit, while HBV accepts all the blame.

## 6. Clinical Implication of Hepatocytic Innate Antiviral Immunity in HDV Infection

The insights gleaned from studies employing PHHs, HLCM-HHs, and HLCM systems suggest that IFN-based antiviral therapy against HDV is unlikely to yield favorable therapeutic outcomes. This is attributed to the IFN refractoriness of infected hepatocytes and the high resistance to the antiviral actions of ISGs. In agreement with these notions, several clinical trials have concluded that antiviral therapy with type I or III IFN lacks definitive clinical efficacy [9,58,82,83]. Moreover, even if the administration of therapeutic IFN achieves undetectable serum HDV RNA at the end of treatment (EOT) and/or end of follow-up (EOFU) [58,84], it remains questionable whether this represents the complete eradication of HDV from infected hepatocytes. This uncertainty arises because HDV is capable of establishing a dormant condition within hepatocytes for an extended period of time, during which the HDV-RNA remains undetectable in the systemic circulation, as demonstrated in studies using HLCM-HHs and the HLCM system [29,57]. In line with this notion, a notable proportion of sustained virological responders at the conclusion of the original clinical trial (either EOT or EOFU) experienced relapse during long-term follow-up [85].

The significance of the hepatocytes’ intrinsic IFN system also extends to our understanding of the viral replication dynamics in HBV–HDV infection in a clinical setting: concurrent HDV infection represses the HBV replication efficiency in a manner analogous to the “paradoxical mode of viral interference”, as discussed in the previous section. Accordingly, it has also been widely recognized that individuals with HDV superinfection exhibit reduced serum HBV-DNA titers compared to those with HBV mono-infection [86,87,88].

The excessive ISG induction and consequent necroinflammation of the liver have been linked to undesirable clinical outcomes, such as liver failure and hepatocellular cancer, as seen in cases with chronic HCV infection [89,90]. Hence, the robust and persistent activation of the hepatic IFN system in HDV infection might provide an explanation, at least in part, for the accelerated disease progression to end-stage liver diseases (ESLDs) such as decompensated cirrhosis and hepatocellular carcinoma. In fact, chronic HBV–HDV co-infection results in the development of ESLDs within 5–10 years, whereas chronic HBV mono-infection typically requires 40 years to the onset of ESLDs [3,91].

In summary, the accumulated knowledge concerning the role of the IFN system in HDV infection, leveraging highly physiological experimental platforms, has yielded instructive insights to aid our understanding of the clinical manifestation of HDV infection.

## 7. Concluding Remarks

The currently available information collectively provides compelling evidence emphasizing the significance of the hepatic IFN system in HDV infection. However, there still exist substantial knowledge gaps in this regard. In particular, it is entirely elusive which and how the cytosolic PRRs, MDA5 and/or RIG-I, sense vRNA species of HDV as the genome replication occurs in the nucleus [92]. Another significant area of inquiry is whether HDV activation of PRRs signaling and its consequences serve as a pro- or antiviral response. This, in turn, raises questions about whether the inhibition of the PRR signaling pathway might represent a novel class of therapeutic target for the management of HDV infection.

Recent studies also revealed that the sensitivity to the antiviral actions of ISGs varies substantially among different genotypes, sub-genotypes, and perhaps strains in both HBV and HDV [37,93]. Hence, our current knowledge, built upon the use of limited viral strains and genotypes, is insufficient to draw definitive conclusions. Further investigations are required to attain a generalized understanding of the role of the hepatic IFN system in regulating these pathogens.

Lastly, it is important to decipher the significance of the innate antiviral immunity of hepatocytes in the context of organ-wide innate immunity. The IFNs and other cytokines/chemokines secreted from infected hepatocytes also govern a multitude of inter-cell-type crosstalk with non-parenchymal cells of the liver, as well as the activation of professional innate immune cells such as Kupffer cells, dendritic cells, and NK cells [94]. The activation of these professional innate immune cells is required for effectively mounting adaptive immune responses, which ultimately play larger roles in determining the clinical outcomes, especially in the later course of infection. The importance of this consideration is exemplified by the fact that HDV co-infection elicits a greater magnitude of immune response, leading to the development of severe acute, perhaps fulminant, hepatitis than that observed in superinfection. As described in the preceded section, the extent of the hepatic IFN system activation is greater in the superinfection than the co-infection. This seemingly counterintuitive phenomena could be explained by the fact that anti-HBV cytotoxic T cells are exhausted in chronic infection, rendering the overall immune response less significant in superinfection [95,96].

To this end, further investigations utilizing humanized liver chimeric mice with reconstituted human immune systems, also known as the dual humanized model, are required to determine the impact of the innate antiviral response of hepatocytes on the establishment of T- and B-cell-mediated immunity.

## Abbreviations

Hepatitis delta virus (HDV), hepatitis B virus (HBV), humanized liver chimeric mice (HLCM), primary human hepatocytes (PHHs).
